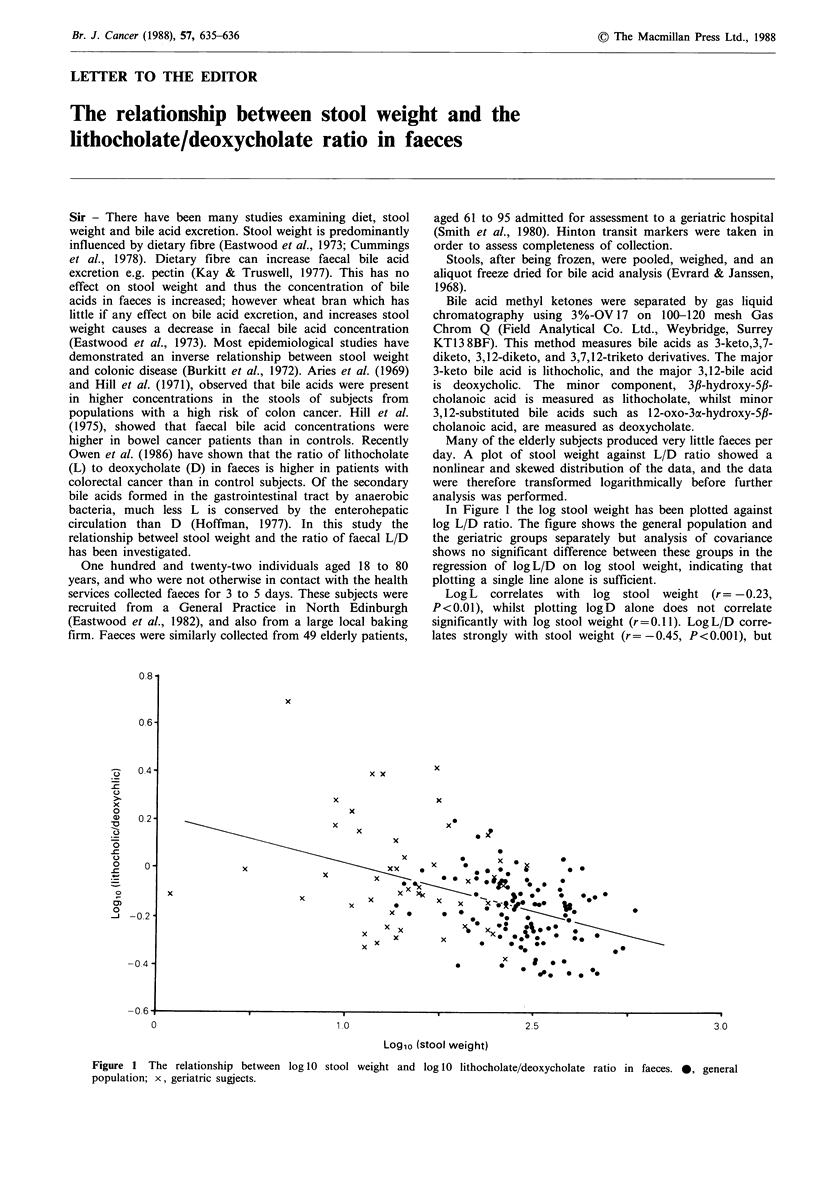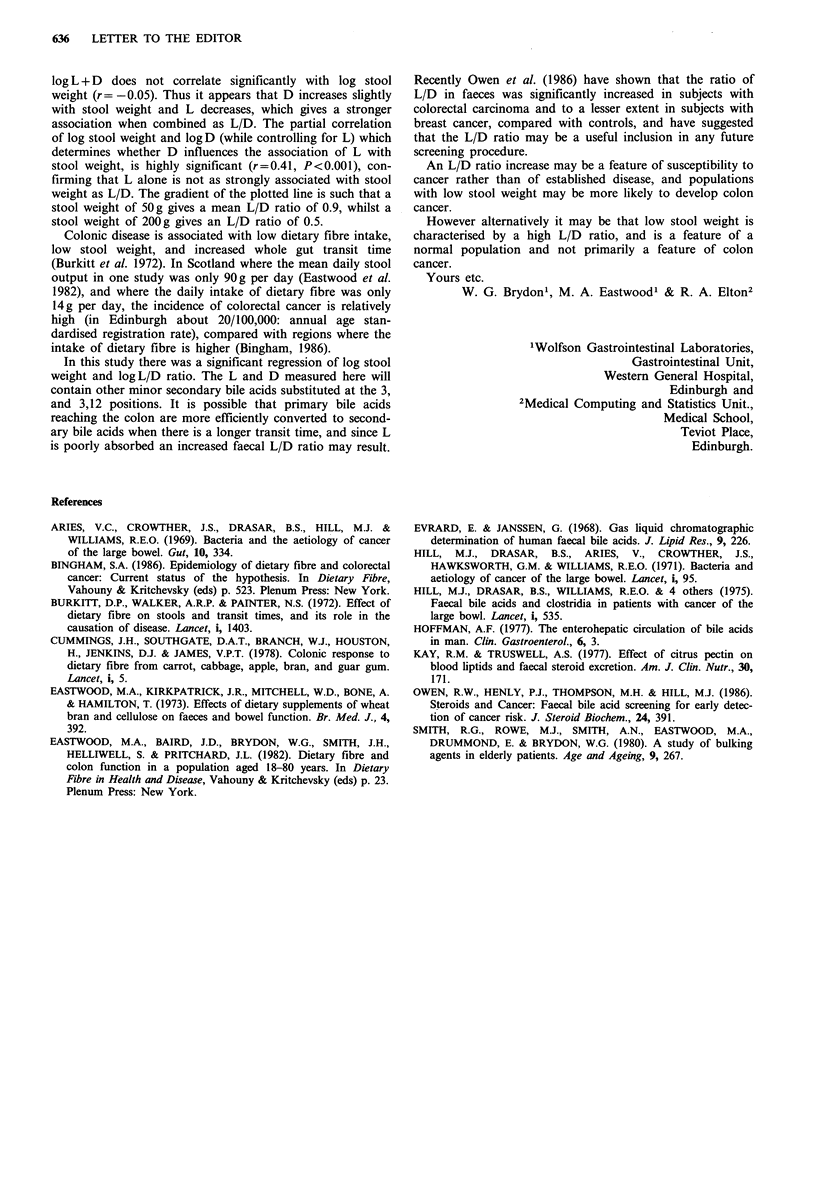# The relationship between stool weight and the lithocholate/deoxycholate ratio in faeces.

**DOI:** 10.1038/bjc.1988.145

**Published:** 1988-06

**Authors:** W. G. Brydon, M. A. Eastwood, R. A. Elton


*Br. J. Cancer* (1988), **57**, 635–636 © The Macmillan Press Ltd., 1988

LETTER TO THE EDITOR

### The relationship between stool weight and the lithocholate/deoxycholate ratio in faeces

**Sir** – There have been many studies examining diet, stool weight and bile acid excretion. Stool weight is predominantly influenced by dietary fibre (Eastwood *et al.*, 1973; Cummings *et al.*, 1978). Dietary fibre can increase faecal bile acid excretion e.g. pectin (Kay & Truswell, 1977). This has no effect on stool weight and thus the concentration of bile acids in faeces is increased; however wheat bran which has little if any effect on bile acid excretion, and increases stool weight causes a decrease in faecal bile acid concentration (Eastwood *et al.*, 1973). Most epidemiological studies have demonstrated an inverse relationship between stool weight and colonic disease (Burkitt *et al.*, 1972). Aries *et al.* (1969) and Hill *et al.* (1971), observed that bile acids were present in higher concentrations in the stools of subjects from populations with a high risk of colon cancer. Hill *et al.* (1975), showed that faecal bile acid concentrations were higher in bowel cancer patients than in controls. Recently Owen *et al.* (1986) have shown that the ratio of lithocholate (L) to deoxycholate (D) in faeces is higher in patients with colorectal cancer than in control subjects. Of the secondary bile acids formed in the gastrointestinal tract by anaerobic bacteria, much less L is conserved by the enterohepatic circulation than D (Hoffman, 1977). In this study the relationship betweel stool weight and the ratio of faecal L/D has been investigated.

One hundred and twenty-two individuals aged 18 to 80 years, and who were not otherwise in contact with the health services collected faeces for 3 to 5 days. These subjects were recruited from a General Practice in North Edinburgh (Eastwood *et al.*, 1982), and also from a large local baking firm. Faeces were similarly collected from 49 elderly patients, aged 61 to 95 admitted for assessment to a geriatric hospital (Smith *et al.*, 1980). Hinton transit markers were taken in order to assess completeness of collection.

Stools, after being frozen, were pooled, weighed, and an aliquot freeze dried for bile acid analysis (Evrard & Janssen, 1968).

Bile acid methyl ketones were separated by gas liquid chromatography using 3%-OV17 on 100–120 mesh Gas Chrom Q (Field Analytical Co. Ltd., Weybridge, Surrey KT13 8BF). This method measures bile acids as 3-keto,3,7-diketo, 3,12-diketo, and 3,7,12-triketo derivatives. The major 3-keto bile acid is lithocholic, and the major 3,12-bile acid is deoxycholic. The minor component, 3$\beta$-hydroxy-5$\beta$-cholanoic acid is measured as lithocholate, whilst minor 3,12-substituted bile acids such as 12-oxo-3$\alpha$-hydroxy-5$\beta$-cholanoic acid, are measured as deoxycholate.

Many of the elderly subjects produced very little faeces per day. A plot of stool weight against L/D ratio showed a nonlinear and skewed distribution of the data, and the data were therefore transformed logarithmically before further analysis was performed.

In Figure 1 the log stool weight has been plotted against log L/D ratio. The figure shows the general population and the geriatric groups separately but analysis of covariance shows no significant difference between these groups in the regression of log L/D on log stool weight, indicating that plotting a single line alone is sufficient.

Log L correlates with log stool weight ($r=-0.23$, $P<0.01$), whilst plotting log D alone does not correlate significantly with log stool weight ($r=0.11$). Log L/D correlates strongly with stool weight ($r=-0.45$, $P<0.001$), but

**Figure 1** The relationship between log 10 stool weight and log 10 lithocholate/deoxycholate ratio in faeces. ●, general population; ×, geriatric sugjects.

log L+D does not correlate significantly with log stool weight ($r=-0.05$). Thus it appears that D increases slightly with stool weight and L decreases, which gives a stronger association when combined as L/D. The partial correlation of log stool weight and log D (while controlling for L) which determines whether D influences the association of L with stool weight, is highly significant ($r=0.41$, $P<0.001$), confirming that L alone is not as strongly associated with stool weight as L/D. The gradient of the plotted line is such that a stool weight of 50 g gives a mean L/D ratio of 0.9, whilst a stool weight of 200 g gives an L/D ratio of 0.5.

Colonic disease is associated with low dietary fibre intake, low stool weight, and increased whole gut transit time (Burkitt *et al.* 1972). In Scotland where the mean daily stool output in one study was only 90 g per day (Eastwood *et al.* 1982), and where the daily intake of dietary fibre was only 14 g per day, the incidence of colorectal cancer is relatively high (in Edinburgh about 20/100,000: annual age standardised registration rate), compared with regions where the intake of dietary fibre is higher (Bingham, 1986).

In this study there was a significant regression of log stool weight and log L/D ratio. The L and D measured here will contain other minor secondary bile acids substituted at the 3, and 3,12 positions. It is possible that primary bile acids reaching the colon are more efficiently converted to secondary bile acids when there is a longer transit time, and since L is poorly absorbed an increased faecal L/D ratio may result. Recently Owen *et al.* (1986) have shown that the ratio of L/D in faeces was significantly increased in subjects with colorectal carcinoma and to a lesser extent in subjects with breast cancer, compared with controls, and have suggested that the L/D ratio may be a useful inclusion in any future screening procedure.

An L/D ratio increase may be a feature of susceptibility to cancer rather than of established disease, and populations with low stool weight may be more likely to develop colon cancer.

However alternatively it may be that low stool weight is characterised by a high L/D ratio, and is a feature of a normal population and not primarily a feature of colon cancer.

Yours etc.

W. G. Brydon[1], M. A. Eastwood[1] & R. A. Elton[2]

[1]Wolfson Gastrointestinal Laboratories,
Gastrointestinal Unit,
Western General Hospital,
Edinburgh and
[2]Medical Computing and Statistics Unit.,
Medical School,
Teviot Place,
Edinburgh.

### References

ARIES, V.C., CROWTHER, J.S., DRASAR, B.S., HILL, M.J. & WILLIAMS, R.E.O. (1969). Bacteria and the aetiology of cancer of the large bowel. *Gut*, **10**, 334.

BINGHAM, S.A. (1986). Epidemiology of dietary fibre and colorectal cancer: Current status of the hypothesis. In *Dietary Fibre*, Vahouny & Kritchevsky (eds) p. 523. Plenum Press: New York.

BURKITT, D.P., WALKER, A.R.P. & PAINTER, N.S. (1972). Effect of dietary fibre on stools and transit times, and its role in the causation of disease. *Lancet*, **i**, 1403.

CUMMINGS, J.H., SOUTHGATE, D.A.T., BRANCH, W.J., HOUSTON, H., JENKINS, D.J. & JAMES, V.P.T. (1978). Colonic response to dietary fibre from carrot, cabbage, apple, bran, and guar gum. *Lancet*, **i**, 5.

EASTWOOD, M.A., KIRKPATRICK, J.R., MITCHELL, W.D., BONE, A. & HAMILTON, T. (1973). Effects of dietary supplements of wheat bran and cellulose on faeces and bowel function. *Br. Med. J.*, **4**, 392.

EASTWOOD, M.A., BAIRD, J.D., BRYDON, W.G., SMITH, J.H., HELLIWELL, S. & PRITCHARD, J.L. (1982). Dietary fibre and colon function in a population aged 18–80 years. In *Dietary Fibre in Health and Disease*, Vahouny & Kritchevsky (eds) p. 23. Plenum Press: New York.

EVRARD, E. & JANSSEN, G. (1968). Gas liquid chromatographic determination of human faecal bile acids. *J. Lipid Res.*, **9**, 226.

HILL, M.J., DRASAR, B.S., ARIES, V., CROWTHER, J.S., HAWKSWORTH, G.M. & WILLIAMS, R.E.O. (1971). Bacteria and aetiology of cancer of the large bowel. *Lancet*, **i**, 95.

HILL, M.J., DRASAR, B.S., WILLIAMS, R.E.O. & 4 others (1975). Faecal bile acids and clostridia in patients with cancer of the large bowl. *Lancet*, **i**, 535.

HOFFMAN, A.F. (1977). The enterohepatic circulation of bile acids in man. *Clin. Gastroenterol.*, **6**, 3.

KAY, R.M. & TRUSWELL, A.S. (1977). Effect of citrus pectin on blood liptids and faecal steroid excretion. *Am. J. Clin. Nutr.*, **30**, 171.

OWEN, R.W., HENLY, P.J., THOMPSON, M.H. & HILL, M.J. (1986). Steroids and Cancer: Faecal bile acid screening for early detection of cancer risk. *J. Steroid Biochem.*, **24**, 391.

SMITH, R.G., ROWE, M.J., SMITH, A.N., EASTWOOD, M.A., DRUMMOND, E. & BRYDON, W.G. (1980). A study of bulking agents in elderly patients. *Age and Ageing*, **9**, 267.